# The Predictive Value of Golgi Protein 73 in Differentiating Benign from Malignant Liver Tumors

**DOI:** 10.1371/journal.pone.0100187

**Published:** 2014-07-17

**Authors:** Mirelle E. E. Bröker, Jan N. M. Ijzermans, Caroline D. M. Witjes, Hanneke J. van Vuuren, Robert A. de Man

**Affiliations:** 1 Department of Surgery, Erasmus Medical Center, Rotterdam, The Netherlands; 2 Department of Gastroenterology and Hepatology, Erasmus Medical Center, Rotterdam, The Netherlands; Drexel University College of Medicine, United States of America

## Abstract

**Introduction:**

In the work up of primary solid liver lesions it is essential to differentiate correctly between benign and malignant tumors, such as hepatocellular adenoma (HCA) and hepatocellular carcinoma (HCC) respectively. A promising new marker to detect HCC is Golgi Protein 73 (GP73). Studies comparing patients with HCC and cirrhosis with normal controls suggested that GP73 is specific for patients with HCC; however, patients with other liver tumors were not included. We therefore studied the predictive value of GP73 in differentiating between solid benign and malignant liver tumors.

**Materials and Methods:**

This study included 264 patients: 88 patients with HCC, 88 with hepatocellular adenoma (HCA), and 88 with focal nodal hyperplasia (FNH). A blood sample was collected from each patient to measure GP73 levels using a quantitative ELISA assay and differences in outcome between subgroups were compared. The receiver operating characteristic (ROC) curve, sensitivity and specificity of GP73 were calculated and compared to alpha-fetoprotein (AFP) levels.

**Results:**

When comparing malignant and benign liver tumors the area under ROC was 0.701 and 0.912 for GP73 and AFP respectively. Test characteristics revealed a sensitivity of 60% for GP73 and 65% for AFP; in addition the specificity was 77% for GP73 and 96% for AFP.

**Conclusion:**

Although the literature suggests that GP73 is a valuable serum marker in patients with HCC, the serum concentration may also be increased in patients with solid benign liver tumors. Therefore, a GP73 assay is less suitable for discriminating between primary malignant and benign tumors of the liver.

## Introduction

Over the past 15 years ultrasound examination of the liver has increased in frequency [1], [2]. Ultrasonography can be used to detect solid liver lesions in asymptomatic patients [3]. Unfortunately, such a finding may cause distress when additional characterization is unable to differentiate between a benign liver tumor, such as hepatocellular adenoma (HCA) and focal nodular hyperplasia (FNH), and a malignant tumor such as hepatocellular carcinoma (HCC). Even when more refined imaging technologies are used, such as contrast-MRI or contrast-enhanced ultrasound, a definitive diagnosis may be difficult to establish in such solid ‘incidentaloma’ in the liver [4], eventually leading to a liver biopsy.

Although in some cases it may be possible to differentiate between malignant and benign tumors using molecular markers, the low sensitivity of tests to detect HCC via serum alpha-fetoprotein (AFP) limits clinical decision making [5]. Therefore more accurate markers are needed. Golgi Protein 73 (GP73), also named Golgi phosphoprotein 2 (GOLPH2), was recently introduced as a potential new candidate to identify HCC. GP73 is a resident Golgi-specific membrane expressed by biliary epithelial cells and is enhanced in HCC cells [6].

Several studies have described GP73 as a HCC-specific marker. However, these studies mainly included patients with liver cirrhosis and/or healthy people as controls [7], [8], and thus lack information on patients with other liver tumors such as HCA and FNH. We therefore determined whether GP73 can differentiate between solid benign and malignant liver tumors and whether GP73 has a predictive value if an unknown solid liver ‘incidentaloma’ is present.

## Materials and Methods

Study protocol was in conformity with the ethical guidelines of the 1975 Declaration of Helsinki and approved by the local Institutional Review Board and Ethical Committee from the Erasmus MC University. Oral informed consent was obtained from all patients, as was approved by the Institutional Review Board. The no-objection policy was approved as only one extra blood vial was collected during regular blood sampling and patients were informed prior to blood sampling. All patients visiting the out-patient department are informed before they visit the clinic that data generated from their visit can be used for scientific studies as we are an academic hospital. Patients can actively opt out when visiting the out-patient clinic. This no-objection procedure has been approved for all outpatient visits. The protocol was approved separately. Blood sampling and the purpose of it are discussed with the patients during their visit at the outpatient clinic as, according to Dutch law, patients have to be informed for which purpose blood samples are taken.

Between July 2007 and October 2012 a total of 264 patients enrolled in this study: 88 patients with HCA, 88 patients with FNH and 88 patients with HCC.

Patients aged 18 years and older, with a proven diagnosis of hepatocellular carcinoma, hepatocellular adenoma or focal nodular dysplasia, were included. The diagnosis was based on histopathology (95 patients, 36%), and if histopathology was not available, on two imaging modalities (magnetic resonance imaging, computed tomography or contrast enhanced ultrasound). All patients had been discussed by our multidisciplinary tumor board committee. Patients were excluded if there was doubt about the diagnosis or if multiple types of tumor were present in the liver.

Data characteristics and a 10-ml blood sample were collected from each patient in the out-patient clinic of the Erasmus University Medical Center. Each blood sample was centrifuged and the serum aliquotted and stored at −80°C until tested.

Blood samples were blinded for analysis. Quantitative ELISA (Antibodies-online GmH, Germany, ABIN365730, intra-assay CV% less than 8%, inter-assay CV% less than 10%.) was performed to measure GP73 levels according to the manufacturer’s instructions. A standard curve was run for each assay using six provided standards, measured in duplicate per ELISA.

The serum AFP level was also determined for each patient using the Elecsys AFP quantitative electrochemiluminescence immunoassay (Roche, Switzerland) and a value >10 µg/L was considered as an elevated level. The receiver operating characteristic (ROC) curve, sensitivity and specificity, and positive predictive value of GP73 were calculated and compared with those of AFP. To determine the optimal cut-off value for GP73, ROC was constructed using all possible cut-offs for each essay. The area under the curve (AUC) was constructed for both AFP and GP73, including 95% confidence intervals (CI). Approval was obtained from the medical ethics committee.

Our primary hypothesis was that GP73 was superior to the predictive value of AFP for the detection of HCC. A power analysis was conducted using a sensitivity of 85% for GP73 and 58% for AFP, with a specificity of 97% and 85%, respectively [9], [10]. A minimum of 40 patients per group was needed for an alpha of 0.05 and a beta of 0.20. The number of patients was increased to the maximum number of wells on the ELISA plates (N = 88).

### Data analysis

Variables were compared using the t-test or a one-way ANOVA, whenever appropriate. Statistical significance was considered at a p-value<0.05. All analyses were performed using the Statistical Package for the Social Sciences (SPSS) (IBM Corp. Released 2011. IBM SPSS Statistics for Windows, Version 20.0. Armonk, NY: IBM Corp). Patient characteristics and treatment were compared using the t-test, the chi-square-test and the Fisher exact test whenever appropriate.

## Results

A total of 264 patients were enrolled in this study, including 88 patients with HCC, 88 with HCA, and 88 with FNH. The demographic and etiologic data of these patients are shown in Table 1. The percentage of males, age, body mass index (BMI), alanine aminotransferase (ALT), aspartate aminotransferase (AST) and gamma-glutamyl transferase (GGT) differed significantly between patients with HCC, HCA and FNH.

**Table 1:** Patient characteristics.

	HCC N = 88	HCA N = 88	FNH N = 88	P-value
Male gender (%)	62 (94%)	2 (3%)	2 (3%)	<0.001
Age (years)	63 (34–82)	40 (20–58)	38 (19–70)	<0.001
BMI (kg/m^2^)	27 (17–39)	30 (19–62)	26 (16–37)	<0.001
AST (I/U)	82 (15–897)	30 (13–86)	26 (8–64)	<0.001
ALT (I/U)	61 (8–461)	33 (7–126)	26 (5–121)	<0.001
GGT (I/U)	304 (11–4570)	88 (8–802)	67 (11–372)	<0.001
Lesion size (mm)	65 (10–250)	61 (8–177)	54 (4–110)	0.178
HBV	16 (18%)	-	-	-
HCV	15 (17%)	-	-	-

Data are presented as median (range) unless other indicated.

HCC: hepatocellular carcinoma, HCA: hepatocellular adenoma, FNH: focal nodular hyperplasia, BMI: body mass index, AST: aspartate aminotransferase, ALT: alanine aminotransferase, GGT HBV: hepatitis B virus, HCV: hepatitis C virus.

**Figure 1:**
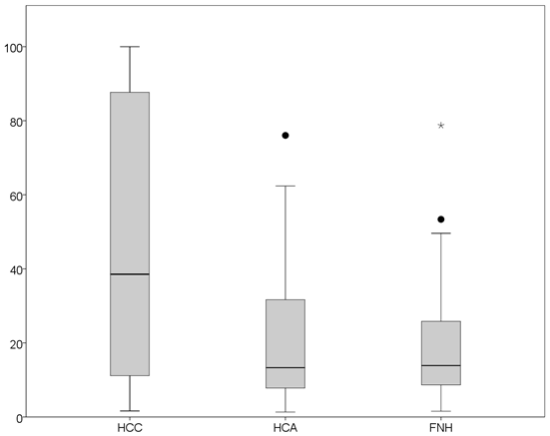
Boxplot GP73. Boxplots showing the serum GP73 levels in patients with hepatocellular carcinoma (HCC), hepatocellular adenoma (HCA) and focal nodular hyperplasia (FNH). •indicates the outliers and *the extreme.

Table 2 shows the distribution of serum GP73 values (IU/l) in the different groups of patients. The mean serum concentration of GP73 was 47 IU/l in the HCC group, 21 IU/L in the HCA group and 17 IU/l in the FNH group (P<0.001). Within the HCC group, GP73 did not differ between patients with hepatitis compared with patients without hepatitis, at 47 and 48 IU/l respectively (p = 0.51). The median serum concentration of AFP was 9184 Ug/L in the HCC group, 3 Ug/L in the HCA group and 3 Ug/L in the FNH group (P = 0.001). The data are shown in Table 2 and Figure 1. In Figure 1 three outliers are depicted (1 HCA, 2 FNH). In the patient with the extreme (FNH) a biopsy was performed. In the two other patients the diagnosis was confirmed by two imaging modalities (magnetic resonance imaging, computed tomography or contrast enhanced ultrasound) in 2010. Follow-up did not reveal a HCC.

**Table 2:** Biomarkers to differentiate between benign and malignant liver tumors.

	HCC	HCA	FNH	P-value
GP73 (IU/ml)	39 (16–100)	13 (1.3–76)	14 (1.6–78)	<0.001
AFP (Ug/L)	9184 (1–212600)	3 (1–16)	3 (1–50)	0.001

Data are presented as median (range).

HCC: hepatocellular carcinoma, HCA: hepatocellular adenoma, FNH: focal nodular hyperplasia, GP73: Golgi Protein 73, AFP: alpha-fetoprotein.

### ROC curves

ROC curves were plotted to determine the optimal cut-off value for GP73 and to identify the sensitivity and specificity of GP73 and AFP in differentiating patients with malignant and benign solid liver tumors (HCC vs. HCA and FNH). The AUC for GP73 was 0.701 with a 95% CI of 0.625 to 0.776, and a sensitivity of 60% and specificity of 77%, using a cut-off value of 29.2 IU/L. The positive predictive value (PPV) for GP73 was 56% and the accuracy of the test was 71%.

**Figure 2:**
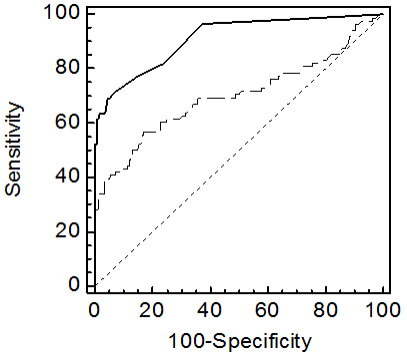
ROC-Curve GP73 and AFP. ROC curves comparing AFP (straight line) and GP73 (dashed line). Pairwise comparison of ROC curves (Hanley & McNeil, 1983) revealed a significant difference of P<0.0001.

The AUC for AFP was 0.91 (95% CI of 0.871 to 0.943); with a cut-off value of 10 Ug/L, the sensitivity was 77% and the specificity was 96%. The PPV for AFP was 89% with an accuracy of 85%. Comparing the two ROC curves showed AFP to be superior to GP73 (p<0.001) (Fig. 2).

Using a cut-off value of 2.92 IU/L, GP73 was positive in 17 out of 31 AFP-negative HCC patients. AFP and GP73 were combined and were reported as positive if one out of two markers, AFP or GP73, was positive. The sensitivity and specificity of the combined marker was 84% and 73%, respectively (Table 3).

**Table 3:** Sensitivity and specificity of the biomarkers.

	Sensitivity	Specificity
GP73	60%	77%
AFP	77%	96%
AFP combined with GP73	84%	73%

GP73: Golgi Protein 73, AFP: alpha-fetoprotein.

## Discussion

When a solid tumor of unknown origin is found in the liver an extensive diagnostic work-up is often necessary. In 10–40% of cases the final diagnosis remains unclear unless invasive techniques are used [4]. Therefore it is of utmost importance to find a serological marker, with a high sensitivity and specificity that is able to discriminate between benign and malignant solid liver tumors. Recent studies showed the potential of GP73 as a marker for HCC [7], [10]–[12]. They suggested that GP73 might even be better than AFP [10]. This study showed the potential of GP73 to distinguish patients with HCC from patients with a solid benign liver tumor, HCA or FNH. However almost all serological data concerning GP73 and AFP in patients with HCC used patients with cirrhosis, hepatitis or no liver disease as controls [8], [9], [12]–[18].

It has been suggested that GP73 could be increased in liver disease due to viral causes (hepatitis B virus and hepatitis C virus) [19], [20]. GP73 could even be associated with the progression of this liver disease [18]. However no significant difference was found between patients with HCC with hepatitis compared with patients without hepatitis. As there were no patients with hepatitis in the HCA or FNH group, this could not explain the elevated levels of GP73 in patients with HCA or FNH. Riener et al. performed immunohistochemical staining of GP73 on tumor samples from HCC, as well as a small group of tumor samples of HCA and FNH, and found that GP73 is frequently expressed in samples of HCA and FNH [21]. In combination with our results, this suggests that GP73 is not a specific marker for HCC.

Two studies included serum from other focal liver lesions. Tian et al included 6 patients with FNH, in 3 out of 6 patients (50%) serum GP73 was elevated (14). Mao et al. suggested that GP73 might be a useful tool for discriminating benign from malignant liver tumors (9.) Although they only studied a small group of patients with benign liver tumors (hepatic cysts, FNH and hepatic cystadenoma), they also found an elevated serum GP73 in the group of patients with benign liver tumors [9]. Our study, which was conducted in a larger and better-defined group of patients with solid liver tumors, confirmed the significantly higher levels of GP73 in patients with HCC. However, analysis of AFP in our study population indicated that AFP is superior to GP73 for discriminating patients with HCC from patients with a solid benign liver tumor. The low number of patients with a false positive test result for AFP was particularly noticeable.

In recent studies evaluating the value of GP73, three types of assay were used: immunoblot assay, Western blot assay and ELISA. It has been suggested that GP73-specific antibodies might interfere with the ELISA analysis [7], [22], as five studies that used ELISA found no significant elevation of GP73 when comparing the serum levels of HCC patients with their controls [14], [15], [17], [23]. Immunoblot assay is too labor-intensive for large patient numbers [12], therefore ELISA is preferred. As we found a significant difference in GP73 between malignant and benign solid liver tumors, we believe that the use of ELISA is no longer an obstacle for the performance of large-scale studies.

Although the ELISA GP73 test is suitable we do not believe that further testing and development for unknown solid tumors in the liver will lead to better results in patients with a solid liver tumor of unknown origin. We do not expect GP73 to complement the results of AFP, as we studied a large and unique group of patients with benign and malignant solid liver tumors. Therefore imaging will continue to have an important place, next to AFP, in distinguishing benign from malignant liver tumors. If GP73 is further developed and analyzed to determine whether it is able to distinguish patients with hepatitis and cirrhosis from patients with HCC, one should take into account that GP73 is also elevated in benign liver tumors.
